# Resource mobilization for tetanus vaccination in Vietnam: Uptake, demand and willingness to pay among women of reproductive age

**DOI:** 10.3389/fpubh.2022.980850

**Published:** 2022-10-18

**Authors:** Thang Huu Nguyen, Xuan Thi Thanh Le, Long Hoang Nguyen, Huong Thi Le, Toan Thanh Thi Do, Huong Lan Thi Nguyen, Hien Thu Nguyen, Carl A. Latkin, Cyrus S. H. Ho, Roger C. M. Ho

**Affiliations:** ^1^School of Preventive Medicine and Public Health, Hanoi Medical University, Hanoi, Vietnam; ^2^VNU School of Medicine and Pharmacy, Vietnam National University, Hanoi, Vietnam; ^3^Institute for Global Health Innovations, Duy Tan University, Da Nang, Vietnam; ^4^Faculty of Nursing, Duy Tan University, Da Nang, Vietnam; ^5^Bloomberg School of Public Health, Johns Hopkins University, Baltimore, MD, United States; ^6^Department of Psychological Medicine, Yong Loo Lin School of Medicine, National University of Singapore, Singapore, Singapore; ^7^Institute for Health Innovation and Technology (iHealthtech), National University of Singapore, Singapore, Singapore

**Keywords:** tetanus, vaccination, uptake, demand, willingness to pay

## Abstract

**Introduction:**

Tetanus vaccine coverage in Vietnam has been declining in recent years due to a rapid population growth rate, shrinking budget, and inefficient resource mobilization strategy. This study examined the uptake, demand, and willingness to pay (WTP) for tetanus vaccines in Vietnamese women of reproductive age as well as determined associated factors and assessed the feasibility of the long-term tetanus vaccine resource mobilization scheme.

**Methods:**

Cross-sectional data were obtained on 807 women of childbearing age in Hanoi, Vietnam in 2016. Tetanus vaccine uptake, demand, and willingness to pay were collected by using a structured questionnaire. Multivariable logistic and interval regression models were used to examine associated factors with vaccine uptake, demand, and WTP.

**Results:**

Of 807 participants, 42.4 and 64.8% had sufficient tetanus vaccination (i.e., received at least three doses of vaccine) and were willing to pay for tetanus vaccination. The mean amount of WTP for one dose of tetanus was US$ 7.3 (95% CI = 6.7–7.9). Having children or being aware that the tetanus vaccine was free-of-charge were negatively associated with WTP for tetanus vaccine. Having a high school education, living in a rural area, and not being aware of vaccine prices or being aware that vaccines were provided freely reduced the amount of WTP. WTP increased among women receiving information from friends and relatives.

**Conclusion:**

Despite of exemption from the tetanus vaccination programs, this study indicated a low tetanus vaccination coverage and a moderate degree of WTP for tetanus vaccine among Vietnamese women of childbearing age. Target-specific educational and financial support interventions, along with efforts to reduce vaccination costs are critical to improving the vaccine uptake, demand, and WTP for tetanus immunization among women.

## Introduction

Tetanus is an acute condition caused by an exotoxin from *Clostridium tetani* bacterium which enters the body through wounds from tissue injuries (1). An estimate from the Global Burden of Disease project indicated that 56,743 tetanus-related deaths occurred worldwide in 2015 (2) and about over 73,000 total tetanus cases including over 27,000 neonatal tetanus infections in 2019 (3). Another report from the World Health Organization (WHO) suggested that 34,019 deaths were related to neonatal tetanus in the same year (4). The introduction of the tetanus toxoid vaccine dramatically diminishes the incidence of tetanus in developed countries (1). Though preventable by vaccination, this fatal disease remains a great public health challenge in low- and middle-income countries with low vaccination coverage (5). In the coming years, substantial climate changes will give rise to favorable environmental conditions (e.g., extreme weather and natural disasters) for the tetanus outbreak by elevating tetanus-prone wounds or disrupting vaccination programs (6). Therefore, increasing tetanus vaccination accessibility and coverage among pregnant women and women of childbearing age to prevent infant mortality is a high priority of the international and national public health agenda (7).

Globally, several initiatives have been implemented to reduce maternal and neonatal deaths due to tetanus such as the Maternal and Neonatal Elimination Initiative (7), Expanded Program on Immunization (EPI) (7), and the Global Vaccine Action Plan (8). Through these campaigns, 116.2 million infants worldwide (accounting for 85% of infants) were offered three doses of the diphtheria-tetanus–pertussis vaccine (DTP3) in 2017 (9). However, universal coverage requires greater efforts given that ~20 million children have to be vaccinated annually (10). Furthermore, it has been found that not only the diphtheria-tetanus–pertussis vaccine but subsequent booster doses are required in order to achieve optimal antibody levels (11). This expands significantly the target populations and puts heavy pressure on the resources needed for the vaccination program (12), particularly in low-middle income countries [e.g., India, Nigeria, or Indonesia (13)]. To date, major financial sources for the tetanus vaccination program in these countries come from international donors namely the WHO, Bill & Melinda Gates Foundation, and Global Vaccine Alliance-GAVI (8). However, along with economic growth of each country, this funding structure will be changed drastically from international aid to domestic sources such as the government's subsidy or people's co-payments (14).

In Vietnam, there are two primary tetanus vaccination programs including the Expanded Program on Immunization (15) and the Maternal and Neonatal Tetanus (MNT) elimination initiative (16). Despite successfully achieving vaccination targets in the past, the tetanus vaccination coverage is still declining. According to the WHO database, the one dose of DTP (DTP1) and three doses of diphtheria-tetanus–pertussis vaccine coverage reduced from 98 and 94% in 2017 to 78 and 75% in 2018, respectively (17). Vietnam has received financial support from the GAVI to perform the vaccination program; but, like other low-middle income countries, this fund will end in the next decades as Vietnam is evolving into a middle-income nation (18). These challenges motivate the government to find appropriate solutions for mobilization of resources for tetanus vaccination. This study aims to examine the uptake, demand, and willingness to pay for tetanus vaccines in Vietnamese pregnant women or women of childbearing age as well as determine associated factors. This information is critically important to evaluate the feasibility of resource mobilization for the tetanus vaccination program in Vietnam in the longer run.

## Methods

### Study setting and participants

A cross-sectional study was performed in April 2016 in four communes of Hanoi, Vietnam, including: Trung Tu and Phuong Lien communes from Dong Da district (urban setting) and Thuy An and Phong Van communes from Ba Vi district (rural setting). In both districts, the percentage of population was very much migratory. In Ba vi rural district, a big population relied on farming activities. People who were pregnant or having a child aged below 12 months were invited to this study. Other inclusion criteria consisted of 1) living in research settings for at least 6 months and 2) agreeing to join in the study. Participants who had any cognitive impairments or disabilities that might hinder their ability to respond to the questionnaire would be excluded from this study.

Local government assistance was provided to all eligible women in the research areas. They were then picked at random by computer software and called. If they were rejected, we would invite the next person on the list. A total of 807 women were enrolled in the study.

### Measurement

Face-to-face interviews from 20 to 25 min were carried out by trained intensive interviewers, who were medical students at Hanoi Medical University. The questionnaire included questions about:

#### Socio-demographic characteristics

The information about age, education, job, number of children, living area, and ever having been ill during their pregnancy were asked participants. A Household's economic status was divided into five quintiles from “poorest” to “richest” by asking participants to report total household monthly income.

#### Tetanus history, awareness, and vaccination uptake

The information collected in this section included: whether ever had tetanus or not, ever heard about the tetanus vaccine, the source of information, whether being vaccinated against tetanus, and the number of doses of tetanus vaccine received before the interview. Participants were classified into the “Sufficient tetanus vaccination” group if they were vaccinated at least three doses at the time of the interview according to the Law on Infectious Disease Prevention and Control (dated November 21, 2007); otherwise, they were categorized into “Insufficient tetanus vaccination” group. Finally, participants were asked about their demand (Yes/No) for tetanus vaccination in the future.

#### Accessibility of tetanus vaccination service

Participants were asked to report the nearest health facility offering tetanus vaccination, travel time, and distance from home to this facility.

#### Willingness to pay for tetanus vaccine

**Figure 1:**
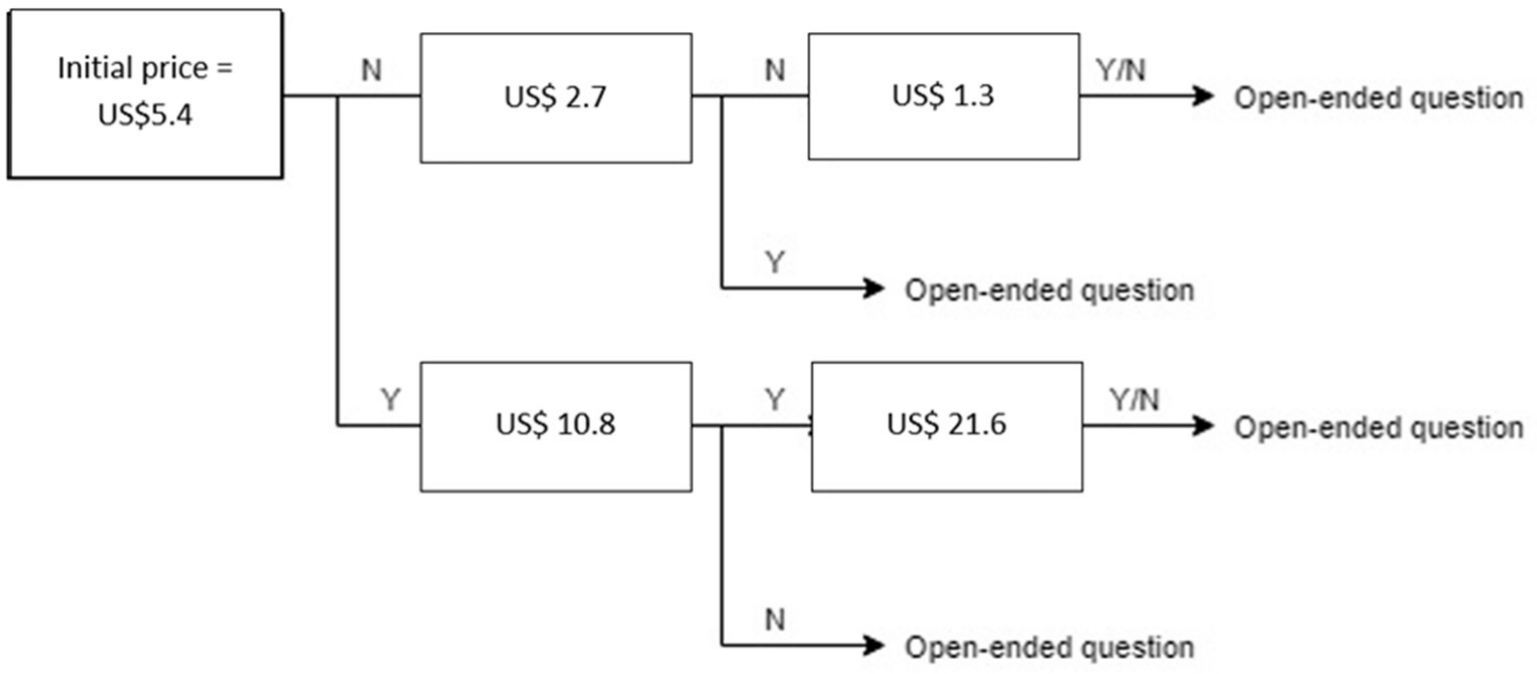
Contingent valuation of willingness to pay. Y, yes; N, no.

In this study, the willingness to pay (WTP) and the amount of willingness to pay for one dose of tetanus vaccination were obtained using a contingent valuation technique using a double-bounded dichotomous choice. The bidding process was showed in Figure 1. The 120,000 VND (or US $5.4, 2019 exchange rate) per one dose of the tetanus vaccine, which was selected based on the real price of the on-demand vaccination service, was used as a first bid for the question about willingness to pay of tetanus vaccine. Then, if they answered “No”, they were asked to decide whether they were willing to pay US$ 2.7, whereas the bid amount was US$ 10.8 if they answered “Yes” for the first bid. At the end of the process, participants were asked to respond to the maximum amount of willingness to pay for one dose of the tetanus vaccine. The amount of willingness to pay for one dose of the tetanus vaccine was calculated by using interval regression.

### Data analysis

STATA version 16.0 (Stata Corp. LP, College Station, United States of America) was used to analyzed the data. We used Chi-squared test to examine the difference in demand for tetanus vaccination among different characteristics regarding socio-demographic, tetanus vaccination history, and accessibility of tetanus vaccination. Multivariable logistic and Interval regression with stepwise forward selection strategies were applied to examine the factors associated with demand and amount of willingness to pay. The variable with *p*-value of < 0.2 were selection for the regression model. If the *p*-value was < 0.05, the result was statistically significant.

### Ethical consideration

The Ethics Committee of Hanoi Medical University was reviewed and approved the study protocol (Code number: 184/HMU-IRB dated 14 November 2015).

## Results

Of 807 participants, 42.4% had sufficient tetanus vaccination (i.e., received at least three doses of vaccine) according to the national guideline. 64.8% had demanded as well as were willing to pay for tetanus vaccination. The mean amount of willingness to pay *P* for one dose of tetanus was US$ 7.3 (95% CI = 6.7–7.9). Table 1 indicated that the rates of sufficient tetanus vaccination were significantly higher in respondents who were aged >30 years (57.2%), had secondary school education or less (56.6%), were farmers (51.1%), had at least one child (46.2%), lived in the rural area (47.8%), and had the lowest monthly household income (52.3%; *p* < 0.05). Meanwhile, the proportion of women who had demanded and were willing to pay for tetanus vaccination was the highest in those having a university education or more (77.4%), having other jobs (77.1%), not having any children (79.3%), living in the urban area (75.4%) and lived in households with middle-income level (75.0%; *p* < 0.05).

**Table 1:** Tetanus vaccination uptake, demand, and WTP by sociodemographic characteristics.

**Characteristics**	**Total**	**Sufficient tetanus**		**Having demand on and WTP**		**Amount of WTP for one**	
		**vaccination**		**for tetanus vaccination**		**dose of tetanus vaccine (US$)**	
	**%**	***n* (%)**	***p*-value**	***n* (%)**	***p*-value**	**Mean**	**95% CI**
**Total**	807 (100.0)	342 (42.4)		523 (64.8)		7.3	6.7–7.9
**Age group**							
<25 years	144 (17.8)	37 (25.7)	<0.01	88 (61.1)	0.38	5.4	4.1–6.8
25–30 years	380 (47.1)	143 (37.6)		255 (67.1)		7.5	6.6–8.5
>30 years	283 (35.1)	162 (57.2)		180 (63.6)		7.9	6.8–9.1
**Education**							
≤ Secondary school	143 (17.7)	81 (56.6)	<0.01	85 (59.4)	<0.01	6.1	4.6–7.5
High school	235 (29.1)	110 (46.8)		126 (53.6)		3.7	2.8–4.6
Vocational training/college	181 (22.4)	59 (32.6)		120 (66.3)		7.9	6.5–9.2
University	248 (30.7)	92 (37.1)		192 (77.4)		11.1	9.8–12.3
**Job**							
Housewife	110 (13.7)	40 (36.4)	<0.01	65 (59.1)	<0.01	7.1	5.3–8.8
Farmer	221 (27.5)	113 (51.1)		117 (52.9)		4.0	3.0–5.0
White-collar	222 (27.6)	83 (37.4)		165 (74.3)		10.1	8.8–11.4
Blue-collar	57 (7.1)	22 (38.6)		32 (56.1)		3.4	1.7–5.2
Business	146 (18.2)	68 (46.6)		105 (71.9)		9.6	8.0–11.1
Others	48 (6.0)	14 (29.2)		37 (77.1)		8.5	5.8–11.3
**Having any children**							
No	82 (10.2)	7 (8.5)	<0.01	65 (79.3)	<0.01	8.2	6.2–10.2
Yes	725 (89.8)	335 (46.2)		458 (63.2)		7.2	6.5–7.9
**Living area**							
Urban area	426 (52.8)	160 (37.6)	<0.01	321 (75.4)	<0.01	10.5	9.6–11.4
Rural area	381 (47.2)	182 (47.8)		202 (53.0)		3.8	3.1–4.6
**Income quintiles**							
Poorest	222 (27.5)	116 (52.3)	<0.01	126 (56.8)	<0.01	5.0	4.0–6.1
Poor	247 (30.6)	98 (39.7)		154 (62.4)		6.5	5.4–7.6
Middle	44 (5.5)	13 (29.6)		33 (75.0)		9.1	6.2–11.9
Rich	201 (24.9)	76 (37.8)		143 (71.1)		9.4	8.0–10.7
Richest	93 (11.5)	39 (41.9)		67 (72.0)		9.6	7.6–11.7

**Figure 2:**
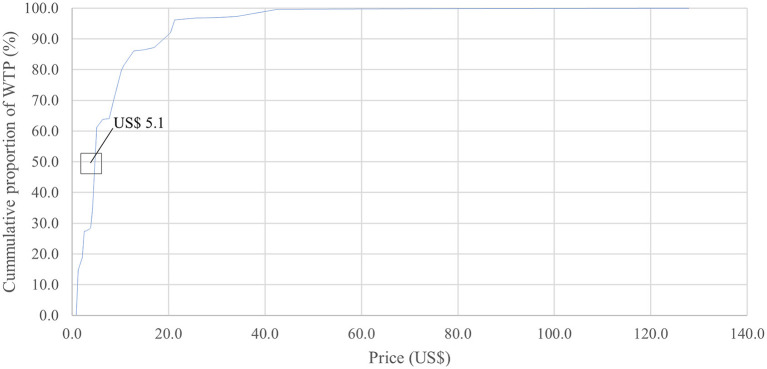
Cumulative proportion of participants' willingness to pay for one dose of tetanus vaccine according to the maximum amount reported.

Figure 2 showed that among our sample who were willing to pay for the tetanus vaccine (64.8%), ~50% sample was willing to pay for an amount of US$ 5.4 (i.e., starting bid) or more per dose. The median maximum price that women were willing to pay was US$ 5.1 per dose.

Table 2 depicts that those ever suffering from illness during pregnancy had also a significantly higher proportion of sufficient tetanus vaccination (55.7%) compared to those without illness during the pregnancy period (41.3%; *p* < 0.05). The rate of sufficient tetanus vaccination and having demand for tetanus vaccination also significantly varied regarding different sources of tetanus vaccination information.

**Table 2:** Tetanus vaccination uptake, demand, and WTP by the history of tetanus and source of tetanus vaccination information.

**Characteristics**	**Total**	**Sufficient tetanus**		**Having demand on and WTP**		**Amount of WTP for one**	
		**vaccination**		**for tetanus vaccination**		**dose of tetanus vaccine (US$)**	
	**%**	***n* (%)**	***p*-value**	***n* (%)**	***p*-value**	**Mean**	**95% CI**
**Ever suffering from illness during pregnancy**							
No	746 (92.4)	308 (41.3)	0.03	483 (64.8)	0.90	7.1	6.4–7.7
Yes	61 (7.6)	34 (55.7)		40 (65.6)		10.0	7.5–12.4
**Ever suffering from tetanus**							
No	780 (97.3)	328 (42.1)	0.46	508 (65.1)	0.31	7.4	6.7–8.0
Yes	22 (2.7)	11 (50.0)		12 (54.6)		4.5	1.3–7.8
**Ever hearing tetanus vaccination**							
No	24 (3.0)	13 (54.2)	0.25	15 (62.5)	0.81	5.9	2.5–9.3
Yes	777 (97.0)	329 (42.3)		504 (64.9)		7.4	6.7–8.0
Source of information*							
School	40 (5.0)	12 (30.0)	0.10	30 (75.0)	0.17	9.4	6.4–12.4
Television	447 (55.4)	191 (42.7)	0.82	262 (58.6)	<0.01	6.1	5.3–7.0
Radio or loudspeaker	161 (20.0)	75 (46.6)	0.23	115 (71.4)	0.049	8.2	6.7–9.7
Newspapers and magazines	175 (21.7)	73 (41.7)	0.84	114 (65.1)	0.92	8.5	7.0–9.9
Internet	327 (40.5)	118 (36.1)	<0.01	240 (73.4)	<0.01	9.6	8.6–10.7
Health provider	449 (55.6)	213 (47.4)	<0.01	298 (66.4)	0.30	7.1	6.2–7.9
Friends and relatives	180 (22.3)	56 (31.1)	<0.01	131 (72.8)	0.01	10.5	9.0–11.9

^*^Compared to “No” group.

The rate of sufficient tetanus vaccination was the highest among women who considered the commune health center the nearest facility offering tetanus vaccination service (47.0%) and had a travel time of fewer than 10 min to the nearest vaccination facility (46.8%). Meanwhile, the lowest proportion of sufficient tetanus vaccination was among women whose homes were more than 5 km far from the nearest vaccination facility (22.7%; *p* < 0.05). The demand for tetanus vaccination significantly differed among groups regarding the nearest facility offering tetanus vaccination, travel time and distance to this facility, as well as the awareness of tetanus vaccine price (*p* < 0.05; Table 3).

**Table 3:** Tetanus vaccination uptake, demand, and WTP by accessibility to tetanus vaccination.

**Characteristics**	**Total**	**Sufficient tetanus**		**Having demand on and WTP**		**Amount of WTP for one**	
		**vaccination**		**for tetanus vaccination**		**dose of tetanus vaccine (US$)**	
	**%**	***n* (%)**	***p*-value**	***n* (%)**	***p*-value**	**Mean**	**95% CI**
**A nearest facility offering tetanus vaccination**							
Commune health center	579 (72.0)	272 (47.0)	<0.01	357 (61.7)	0.02	6.1	5.4–6.8
Public hospital/medical center	98 (12.2)	35 (35.7)		73 (74.5)		10.3	8.4–12.3
Vaccination service center	94 (11.7)	22 (23.4)		69 (73.4)		10.9	8.9–12.8
Other	33 (4.1)	13 (39.4)		21 (63.6)		9.8	6.4–13.2
**Travel time to the nearest vaccination facility**							
≤ 10 mins	477 (59.3)	223 (46.8)	<0.01	308 (64.6)	<0.01	7.5	6.6–8.3
11–20 mins	239 (29.7)	97 (40.6)		170 (71.1)		7.3	6.2–8.5
>20 mins	88 (11.0)	21 (23.9)		44 (50.0)		6.5	4.6–8.4
**Distance from home to the nearest vaccination facility**							
<1 km	130 (16.1)	62 (47.7)	<0.01	79 (60.8)	<0.01	7.3	5.7–8.9
1– <2 km	209 (25.9)	100 (47.9)		157 (75.1)		8.5	7.2–9.8
2– <5 km	349 (43.3)	153 (43.8)		220 (63.0)		6.3	5.4–7.2
≥5 km	119 (14.8)	27 (22.7)		67 (56.3)		8.2	6.4–9.9
**Awareness of tetanus vaccine price**							
Yes	123 (15.3)	46 (37.4)	0.45	103 (83.7)	<0.01	12.2	10.5–13.8
No	424 (52.8)	181 (42.7)		310 (73.1)		8.1	7.2–9.0
Free-of-charge	256 (31.9)	113 (44.1)		107 (41.8)		3.7	2.7–4.6

The results of multivariable regression models are shown in Table 4. Only variables with a *p*-value of < 0.2 were presented. Age, having any children and ever suffering from illness during pregnancy, and receiving information from health providers were facilitators for the sufficient tetanus vaccination among our sample. Having vocational training/college education (OR = 0.51, 95% CI = 0.30–0.87) and more than five km of distance from home to the nearest vaccination facility (OR = 0.43, 95% CI = 0.24–0.78) were reducing sufficient tetanus vaccination uptake significantly.

**Table 4:** Associated factors with tetanus vaccination uptake, demand, and WTP.

**Characteristics**	**Sufficient tetanus**		**Having demand on and WTP**		**Amount of WTP for one**	
	**vaccination**		**for tetanus vaccination**		**dose of tetanus vaccine (US$)**	
	**OR**	**95% CI**	**OR**	**95% CI**	**Coef**.	**95% CI**
**Age**	1.10***	1.06; 1.14				
**Education**						
≤ Secondary school	Ref		Ref		Ref	
High school	0.75	0.47; 1.21	0.72	0.45; 1.16	−2.74***	−4.50; −0.99
Vocational training/college	0.51**	0.30; 0.87	0.91	0.53; 1.57	−0.48	−2.44; 1.47
University	0.69	0.40; 1.19	1.33	0.74; 2.39	0.66	−1.40; 2.73
**Living area**						
Urban area	Ref				Ref	
Rural area	1.40	0.93; 2.11			−4.21***	−5.90; −2.53
**Having any children**						
No	Ref		Ref			
Yes	7.39***	3.10; 17.58	0.43***	0.23; 0.81		
**Ever suffering from illness during pregnancy**						
No	Ref					
Yes	1.83**	1.01; 3.32				
**Awareness of tetanus vaccine price**						
Yes			Ref		Ref	
No			0.61*	0.35; 1.08	−2.01**	−3.78; −0.23
Free-of-charge			0.15***	0.08; 0.28	−3.51***	−5.73; −1.29
**Distance from home to the nearest vaccination facility**						
<1 km	REF		REF		REF	
1– <2 km	1.17	0.72; 1.91	2.51***	1.47; 4.29	2.54***	0.67; 4.42
2– <5km	0.99	0.63; 1.55	1.21	0.73; 2.01	0.01	−1.72; 1.73
≥5 km	0.43***	0.24; 0.78	0.59	0.27; 1.26	−0.14	−2.28; 2.00
**Travel time to the nearest vaccination facility**						
≤ 10 mins			Ref			
11–20 mins			1.40	0.90; 2.19		
>20 mins			0.70	0.35; 1.42		
**Source of information (Yes vs. No)**						
Television			0.68**	0.48; 0.95	−1.50**	−2.71; −0.30
Newspapers and magazines			0.57**	0.37; 0.88		
Internet			1.43*	0.95; 2.14		
Health provider	1.53***	1.12; 2.10				
Friends and relatives	0.63**	0.42; 0.93	1.41	0.92; 2.18	2.24***	0.80; 3.68

^***^p < 0.01; ^**^p < 0.05; ^*^p < 0.1.

OR, Odds Ratio; CI, Confidence Interval; Coef., Coefficient; ref, reference group.

Having any children (OR = 0.43, 95% CI = 0.23–0.81) or being aware that the tetanus vaccine was free-of-charge (OR = 0.15, 95% CI = 0.08–0.28) were negatively associated with having demand on and being willing to pay for a tetanus vaccine. Women whose homes were one to <2 km away from vaccination facilities were more likely to be willing to pay for tetanus vaccination (OR = 2.51, 95% CI = 1.47–4.29). Having a high school education, living in a rural area, and not being aware of vaccine prices or being aware that vaccines were provided freely reduced the amount of WTP. Meanwhile, the amount of WTP increased among women having homes that were from one to <2 km from home to the vaccination facility, as well as those receiving information from friends and relatives (Coef. = 2.24; 95% CI = 0.80–3.68).

## Discussion

To our knowledge, this is among the first studies to investigate tetanus vaccination demand and willingness to pay among women of childbearing age in Vietnam. Our study indicated an unsatisfactorily low tetanus vaccination coverage in this target population. However, more than half of the women showed their demand for tetanus vaccination and were willing to pay for this service. Findings of this study also revealed associated factors with tetanus vaccine uptake, demand, and willingness to pay regarding socio-demographic and service accessibility characteristics, suggesting further implications in mobilizing resources for tetanus vaccination in Vietnam.

Results of this study revealed a large proportion of women of childbearing age in rural and urban areas of Vietnam were immunized to tetanus. In addition to a substantial reduction of the one dose of diphtheria-tetanus-pertussis vaccine coverage reported by the WHO among Vietnamese infants (17). This finding underlines an emerging problem that many infants might lose opportunities to prevent neonatal tetanus if none of the actions would be done. This rate in our study was significantly lower than that in developed countries such as the United States (61–81.6%) (19, 20) and Germany (73.1%) (21), but higher than developing nations such as Ivory Coast (17.8%) (22). Possible explanations for these discrepancies include the differences in study settings, vaccination culture, target populations, and standards to identify optimal tetanus vaccination or health system characteristics (23, 24). For example, in this study, we used the Ministry of Health's standard to determine the sufficient tetanus vaccination, while other studies in Germany, the United States, Spain, and France defined that people having the last shot of tetanus vaccine <10 years ago were sufficiently vaccinated (25–27). Our study also observed that higher age and having children and experiencing illness during pregnancy were associated with tetanus vaccine uptake, which differed from studies in wealthy countries (27, 28). Thus, improving vaccine uptake should require specific interventions focusing on the younger age group, those not having any children, or those not having any illness during the pregnancy period.

Our study was consistent with previous studies that health providers were a pervasive source of information about vaccination (21, 29), and this was a facilitator for tetanus vaccine uptake (21). Indeed, among women of childbearing age, vaccine safety for themselves and their fetuses is the most concerning problem. Previous studies have indicated that vaccine hesitancy was linked to gaps in belief and trust in vaccine safety. Most associated hesitancy toward vaccination were also identified, including: serious immediate reactions, autism and dysfunctioning of the immune system (30, 31). However, scientific evidence suggested that the occurrence rates of these complications were generally or close to none (32–34). Therefore, receiving scientifically accurate information from health professionals would increase their knowledge about potential benefits and risks of tetanus vaccination, and motivate them to get vaccinated (35). A study in Nigeria indicated that missed tetanus immunization among women was strongly correlated with the physician referral for vaccination (36). Notably, we found that participants concerning friends or relatives as an information source were less likely to uptake sufficient vaccines. In literature, friends or family members are important components that can significantly influence the decision for vaccination among women (37). Therefore, along with improving the knowledge of healthcare workers about vaccines for women of childbearing age, it is vital to include women's social elements when designing interventions to enhance vaccine uptake.

We found that two-thirds of our sample were willing to pay for the tetanus vaccine, with the mean and median amount of willingness to pay at US$ 7.3 and US$ 5.1 per dose, respectively (or ~US$ 22 per three doses). This result was lower than that in a study in the United States, which indicated that 72% of adults were willing to pay for tetanus vaccine if the price increased from US$ 25 to US$ 30 (38). Most common reasons for unwillingness to pay included “Not necessary” and “Not at risk of tetanus”. This rate of willingness to pay was somewhat similar to the willingness to pay for other vaccines such as Human papillomavirus vaccines or dengue vaccines in the previous studies in Vietnam (39, 40). Initially, we supposed that these individuals were women who had sufficient tetanus immunization or received at least three doses of the vaccine according to the recommendation of the Ministry of Health. However, after performing additional analysis, we found that ~57% of women who had insufficient immunization did not have the demand for as well as were unwilling to pay for tetanus vaccination. The tetanus vaccine in Vietnam can be approached *via* two routes: free-of-charge *via* the Expanded Program on Immunization and Maternal and Neonatal Tetanus programs or out-of-pocket payments *via* the on-demand vaccination service centers. Most of our sample were not aware of the tetanus vaccine price or believed that this vaccine should be provided freely; thus, they were not willing to pay for the tetanus vaccine. The results of our multivariable regression models confirmed our point of view. Moreover, almost all participants used television as an information source, and they were less likely to be willing to pay and were willing to pay less than those not using television for seeking information. This can be justified that most of the vaccine-related information on television promotes vaccine uptake *via* the Expanded Program on Immunization program, thus influencing their willingness to pay decision. Additionally, rural women were willing to pay less than urban ones, which might be explained by the fact that the economic status of the former women was lower than the latter (41). Interventions to inform the burden of tetanus, the benefits of immunization, and the actual price of vaccination *via* mass media, as well as financial support for economically vulnerable populations, would be effective solutions to increase the willingness to pay for tetanus vaccination. The correlation trend between income and sufficient vaccination indicated that those with lower income had access to free vaccination and the wealthier population were able to pay for tetanus vaccine, meaning those who fall between these groups, specifically women with medium income were least likely to seek or receive care due to financial burden posed by vaccination costs. In our study, the willingness to pay among women equaled to only approximately half of actual price. Therefore, to promote vaccination among medium-income women, the cost of tetanus vaccine should be reduced, such as through higher insurance coverage or national reduction in vaccination fee for a certain income group.

Our study has several limitations that should be noted. First, the cross-sectional design could not allow us to draw the causal relationships between tetanus vaccine uptake, demand, and willingness to pay with associated factors. Second, information about vaccine uptake was recalled which might result in recall bias. Third, despite a large sample size, our sample might not represent the target population in other regions of Vietnam. Finally, given the importance of theoretical framework for explaining behaviors such as vaccination uptake, further studies should be warranted to use behavioral theories to explore the factors associated with vaccination uptake and willingness to pay for vaccination.

## Conclusions

This study indicated a low tetanus vaccination coverage and a moderate degree of willingness to pay for tetanus vaccine among Vietnamese women of childbearing age. Further, target-specific educational and financial support interventions, along with efforts to reduce vaccination costs are critical to improving the vaccine uptake, demand, and willingness to pay for tetanus immunization in these women.

## Data availability statement

The original contributions presented in the study are included in the article/supplementary material, further inquiries can be directed to the corresponding author.

## Ethics statement

The studies involving human participants were reviewed and approved by the Ethics Committee of Hanoi Medical University was reviewed and approved the study protocol (Code number: 184/HMU-IRB dated 14 November 2015). The patients/participants provided their written informed consent to participate in this study.

## Author contributions

Conceptualization: XL, LN, HLTN, and CH. Data curation: HL, TD, and TN. Formal analysis: LN, TD, TN, and CL. Investigation: HL, HTN, and RH. Methodology: HTN, CL, and CH. Supervision: TN, HLTN, CH, and RH. Writing—original draft: XL, HL, TD, and HTN. Writing—review and editing: XL, LN, HLTN, CL, and RH. All authors contributed to the article and approved the submitted version.

## Funding

The article process charge of this paper is supported by NUS Department of Psychological Medicine (R-177-000-100-001/R-177-000-003-001) and NUS iHeathtech Other Operating Expenses (R-722-000-004-731).

## Conflict of interest

The authors declare that the research was conducted in the absence of any commercial or financial relationships that could be construed as a potential conflict of interest.

## Publisher's note

All claims expressed in this article are solely those of the authors and do not necessarily represent those of their affiliated organizations, or those of the publisher, the editors and the reviewers. Any product that may be evaluated in this article, or claim that may be made by its manufacturer, is not guaranteed or endorsed by the publisher.

## References

[1] Hamborsky J Kroger A. Epidemiology and Prevention of Vaccine-Preventable Diseases, E-Book: The Pink Book. Washington, DC: Public Health Foundation (2015).

[2] Kyu HH Mumford JE Stanaway JD Barber RM Hancock JR Vos T. Mortality from tetanus between 1990 and 2015: findings from the global burden of disease study 2015. BMC Public Health. (2017) 17:179. 10.1186/s12889-017-4111-4 28178973 PMC5299674

[3] Centers for Disease Control Prevention (CDC). Maternal and Neonatal Tetanus is a Public Health Problem in 12 Countries and Tetanus Still Affects People Globally. (2022). Available from: https://www.cdc.gov/globalhealth/immunization/diseases/tetanus/data/fast-facts.html#:~:text=In%202019%2C%20the%20Global%20Burden,doses%20of%20tetanus%20containing%20vaccine (accessed March 25, 2022).

[4] World Health Organization (WHO). Protecting All Against Tetanus: Guide to Sustaining Maternal and Neonatal Tetanus Elimination (MNTE) and Broadening Tetanus Protection for all Populations. Geneva: World Health Organization(2019).

[5] Taylor AM. Tetanus. Cont Educ Anaesth Crit Care Pain. (2006) 6:101–4. 10.1093/bjaceaccp/mkl014

[6] Finkelstein P Teisch L Allen CJ Ruiz G. Tetanus: a potential public health threat in times of disaster. Prehosp Disaster Med. (2017) 32:339–42. 10.1017/S1049023X17000012 28215195

[7] World Health Organization (WHO). Expanded Programme on Immunization. 2nd ed. Geneva: World Health Assembly (1989). 42 p.

[8] World Health Organization (WHO). Global Vaccine Action Plan 2011–2020. Geneva: World Health Organization (2013).

[9] World Health Organization (WHO). World Health Statistics 2016: Monitoring Health for the SDGs Sustainable Development Goals. Geneva: World Health Organization (2016).

[10] World Health Organization (WHO). Meeting of the Strategic Advisory Group of Experts on Immunization, April 2017—Conclusions and Recommendations. Switzerland: World Health Organization (2017), p. 301–320.

[11] Aboud S Matre R Lyamuya EF Kristoffersen EK. Levels and avidity of antibodies to tetanus toxoid in children aged 1–15 years in Dar es Salaam and Bagamoyo, Tanzania. Ann Trop Paediatr. (2000) 20:313–22. 10.1080/02724936.2000.11748153 11219170

[12] World Health Organization (WHO). Immunization Supply Chain and Logistics. Geneva: World Health Organization (2014).

[13] Yen LM Thwaites CL. Tetanus. Lancet. (2019) 393:1657–68. 10.1016/S0140-6736(18)33131-3 30935736

[14] Kallenberg J Mok W Newman R Nguyen A Ryckman T Saxenian H. Gavi's transition policy: moving from development assistance to domestic financing of immunization programs. Health Aff. (2016) 35:250–8. 10.1377/hlthaff.2015.1079 26858377

[15] UNICEF. Review of Expanded Program of Immunization Vietnam 2009. National EPI Review Report. New York, NY: UNICEF (2009).

[16] UNFPA UNICEF World Health Organization (WHO). Maternal and Neonatal Tetanus Elimination by 2005: Strategies for Achieving and Maintaining Elimination. Geneva: World Health Organization (2000).

[17] World Health Organization (WHO) UNICEF. WHO-UNICEF Estimates of DTP1 and DTP3 Coverage. Geneva: World Health Organization (2019). Available online at: http://apps.who.int/immunization_monitoring/globalsummary/timeseries/tswucoveragedtp3.html and http://apps.who.int/immunization_monitoring/globalsummary/timeseries/tscoveragedtp1.html (accessed October 29, 2019).

[18] Jit M Dang TT Friberg I Hoang VM Pham Huy TK Walker N. Thirty years of vaccination in Vietnam: impact and cost-effectiveness of the national expanded programme on immunization. Vaccine. (2015) 33:A233–9. 10.1016/j.vaccine.2014.12.017 25919167 PMC4428532

[19] Healy CM Ng N Taylor RS Rench MA Swaim LS. Tetanus and diphtheria toxoids and acellular pertussis vaccine uptake during pregnancy in a metropolitan tertiary care center. Vaccine. (2015) 33:4983–7. 10.1016/j.vaccine.2015.07.018 26192356

[20] Goldfarb IT Little S Brown J Riley LE. Use of the combined tetanus-diphtheria and pertussis vaccine during pregnancy. Am J Obstetr Gynecol. (2014) 211:299.e1–299.e2995. 10.1016/j.ajog.2014.05.029 24858200

[21] Böhmer MM Walter D Krause G Müters S Gösswald A Wichmann O. Determinants of tetanus and seasonal influenza vaccine uptake in adults living in Germany. Hum Vaccin. (2011) 7:1317–25. 10.4161/hv.7.12.18130 22108034

[22] Yaya S Kota K Buh A Bishwajit G. Antenatal visits are positively associated with uptake of tetanus toxoid and intermittent preventive treatment in pregnancy in Ivory Coast. BMC Public Health. (2019) 19:1467–1467. 10.1186/s12889-019-7847-1 31694607 PMC6836543

[23] Vouking MZ Tadenfok CN Ekani JME. Strategies to increase immunization coverage of tetanus vaccine among women in Sub Saharan Africa: a systematic review. Pan Afr Med J. (2017) 27:25. 10.11604/pamj.supp.2017.27.3.11535 29296160 PMC5745987

[24] Ghosh A Laxminarayan R. Demand- and supply-side determinants of diphtheria-pertussis-tetanus nonvaccination and dropout in rural India. Vaccine. (2017) 35:1087–93. 10.1016/j.vaccine.2016.12.024 28081971 PMC5297340

[25] Gautret P Yong W Soula G Parola P Brouqui P DelVecchio Good M-J. Determinants of tetanus, diphtheria and poliomyelitis vaccinations among Hajj pilgrims, Marseille, France. Eur. J. Public Health. (2009) 20:438–42. 10.1093/eurpub/ckp196 19959614

[26] Guthmann J Fonteneau L Antona D Lévy-Bruhl DJBEH. Diphtheria, tetanus and poliomyelitis immunization coverage in French adults: results of the Health and Social Protection survey, 2002. Bull Epidémiol Hebd. (2007) 2007:51–2.

[27] Miller BL Ahmed F Lu PJ Euler GL Kretsinger K. Tetanus and pertussis vaccination coverage among adults aged ≥18 years: United States, 1999 and 2008. MMWR Morb Mortal Wkly Rep. (2010) 59:1302–6. Available online at: https://pubmed.ncbi.nlm.nih.gov/20948508/20948508

[28] Guthmann JP Fonteneau L Antona D Lévy-Bruhl D. Factors associated with tetanus vaccination coverage in adults in France and with knowledge of vaccination status [Déterminants de couverture vaccinale antitétanique chez l'adulte en France et de connaissance du statut vaccinal]. Med Mal Infect. (2010) 40:560–7. 10.1016/j.medmal.2010.03.009 20400252

[29] Miller BL Kretsinger K Euler GL Lu P-J Ahmed F. Barriers to early uptake of tetanus, diphtheria and acellular pertussis vaccine (Tdap) among adults-United States, 2005–2007. Vaccine. (2011) 29:3850–6. 10.1016/j.vaccine.2011.03.058 21459173

[30] Facciolà A Visalli G Orlando A Bertuccio MP Spataro P Squeri R. Vaccine hesitancy: an overview on parents' opinions about vaccination and possible reasons of vaccine refusal. J Public Health Res. (2019) 8:jphr.2019.1436. 10.4081/jphr.2019.1436 30997357 PMC6444379

[31] Giambi C Fabiani M D'Ancona F Ferrara L Fiacchini D Gallo T. Parental vaccine hesitancy in Italy–results from a national survey. Vaccine. (2018) 36:779–87. 10.1016/j.vaccine.2017.12.074 29325822

[32] Children's Hospital of Philadelphia. Vaccines and Autism. Available online at: https://www.chop.edu/centers-programs/vaccine-education-center/vaccines-and-other-conditions/vaccines-autism

[33] Hviid A Wohlfahrt J Stellfeld M Melbye M. Childhood vaccination and nontargeted infectious disease hospitalization. JAMA. (2005) 294:699–705. 10.1001/jama.294.6.699 16091572

[34] Becerra-Culqui TA Getahun D Chiu V Sy LS Tseng HF. Prenatal tetanus, diphtheria, acellular pertussis vaccination and autism spectrum disorder. Pediatrics. (2018) 142:3. 10.1542/peds.2018-0120 30104424

[35] MacDougall DM Halperin SA. Improving rates of maternal immunization: challenges and opportunities. Hum Vacc Immunotherapeut. (2016) 12:857–65. 10.1080/21645515.2015.1101524 26552807 PMC4962946

[36] Edet EE Ikpeme BM Ndifon WO Oyo-Ita AE. Factors associated with missed opportunities to immunise with tetanus toxoid at a tertiary health institution in Nigeria. Cent Afr J Med. (1998) 44:199–202. 10101419

[37] Wilson RJ Paterson P Jarrett C Larson HJ. Understanding factors influencing vaccination acceptance during pregnancy globally: a literature review. Vaccine. (2015) 33:6420–9. 10.1016/j.vaccine.2015.08.046 26320417

[38] Johnson DR Nichol KL Lipczynski K. Barriers to adult immunization. Am J Med. (2008) 121:S28–35. 10.1016/j.amjmed.2008.05.005 18589065

[39] Tran BX Than PTQ Doan TTN Nguyen HLT Thi Mai H Nguyen THT. Knowledge, attitude, and practice on and willingness to pay for human papillomavirus vaccine: a cross-sectional study in Hanoi, Vietnam. Patient Prefer Adherence. (2018) 12:945–54. 10.2147/PPA.S165357 29881260 PMC5985800

[40] Nguyen LH Tran BX Do CD Hoang CL Nguyen TP Dang TT. Feasibility and willingness to pay for dengue vaccine in the threat of dengue fever outbreaks in Vietnam. Patient Prefer Adherence. (2018) 12:1917–26. 10.2147/PPA.S178444 30288032 PMC6163003

[41] Haile ZT Chertok IRA Teweldeberhan AK. Determinants of utilization of sufficient tetanus toxoid immunization during pregnancy: evidence from the Kenya Demographic and Health Survey, 2008–2009. J Commun Health. (2013) 38:492–9. 10.1007/s10900-012-9638-9 23161213

